# Longitudinal trends in self-reported anxiety. Effects of age and birth cohort during 25 years

**DOI:** 10.1186/s12888-017-1277-3

**Published:** 2017-04-26

**Authors:** Susanna Calling, Patrik Midlöv, Sven-Erik Johansson, Kristina Sundquist, Jan Sundquist

**Affiliations:** 1 Department of Clinical Sciences in Malmö, Center for Primary Health Care Research, Lund University, Malmö, Sweden; 2 Stanford Prevention Research Center, Stanford University, Palo Alto, California, USA; 3 Clinical Research Centre (CRC), Skåne University Hospital, Jan Waldenströms gata 35, 205 02 Malmö, Sweden

**Keywords:** Anxiety, Longitudinal studies, Age factors, Cohort effect

## Abstract

**Background:**

Anxiety has been suggested to increase among young individuals, but previous studies on longitudinal trends are inconclusive. The aim of this study was to analyze longitudinally, the changes over time of prevalence of self-reported anxiety in the Swedish population between 1980/1981 and 2004/2005, in different birth cohorts and age groups.

**Methods:**

A random sample of non-institutionalized persons aged 16–71 years was interviewed every eighth year. Self-reported anxiety was assessed using the question” Do you suffer from nervousness, uneasiness, or anxiety?” (no; yes, mild; yes, severe). Mixed models with random intercepts were used to estimate changes in rates of anxiety (mild or severe) within different age groups and birth cohorts and in males and females separately. In addition to three time-related variables – year of interview, age at the time of the interview, and year of birth –the following explanatory variables were included: education, urbanization, marital status, smoking, leisure time physical activity and body mass index.

**Results:**

Overall prevalence of self-reported anxiety increased from 8.0 to 12.4% in males and from 17.8% to 23.6% in females, during the 25-year follow-up period. The increasing trend was found in all age groups except in the oldest age groups, and the highest increase was found in young adults 16–23 years, with more than a three-fold increase in females, and a 2.5-fold increase in males, after adjustments for covariates.

**Conclusions:**

Between 1980/81 and 2004/05, there was an increasing prevalence of self-reported anxiety in all age groups except in the oldest, which indicates increased suffering for a large part of the population, and probably an increased burden on the health care system. Clinical efforts should focus particularly on young females (16–23 years), where the increase was particularly large; almost one third experienced anxiety at the end of the 25-year follow-up.

## Background

Mental illness including anxiety is one of the leading causes of disability worldwide [1, 2]. In the US, the overall life-time prevalence of anxiety disorders is about 25% [3]. In addition to diagnosed anxiety disorders, unreported or undiagnosed mental illness is a common health problem [4–6]. A particularly vulnerable group seems to be adolescents and young adults, for whom the prevalence rates of self-reported anxiety have increased during the last decades, in Sweden as well as in other developed countries [5, 7–10]. However, population-based studies on time trends of anxiety are scarce and inconclusive [11, 12]. One US study reported stable levels of “psychological distress” between 1997 and 2004 [6], but recent US and Canadian surveys revealed that people born in the oldest and more recently born birth cohorts had higher levels of psychological distress than those cohorts born in mid-century [11]. The present study aimed to analyse the time trends of self-reported anxiety symptoms, measured by one survey question over time. The current question has earlier been reported to be a strong predictor of premature mortality and psychiatric disorders [13].

Studies attempting to analyse longitudinal changes have primarily been based on cross-sectional data, which do not take into account changes in the distribution of age and birth cohort [6, 7, 9]. Such studies could not distinguish whether changes are attributable to an age effect (i.e. differences within individuals) or a cohort effect (i.e. differences among individuals at baseline) [7, 9, 11]. The novelty of the present study was to analyse the trends of self-reported anxiety and to disentangle age and cohort effects, by using continuous data of the same individuals for several assessments over a 25-year time period, which will shed light on potential longitudinal changes. Moreover, anxiety is associated with several other individual factors, such as smoking [14], education level [15], obesity [16], and physical activity [17], which may confound the results.

The aim of the present study was to analyze longitudinal trends in self-reported anxiety within different groups of age and birth cohort, in the Swedish population between 1980/81 and 2004/05, by using a mixed model with random intercept. Another aim was to analyze whether any observed effects remained after adjustment for possible confounders/effect modifiers, such as education, urbanization, marital status, and lifestyle factors.

## Methods

### The Swedish Annual Level of Living Survey

We used data from the Swedish Annual Level of Living Survey (SALLS), which has been conducted annually since 1974 by Statistics Sweden, the Swedish government-owned bureau of statistics. The survey comprises a representative, simple cross-sectional random sample of non-institutionalized individuals aged 16–84 years, drawn systematically by age group from the Swedish Total Population Register [18], and the sample represents the entire population of Sweden. In this study, we included 2728 males and 2770 females aged 16–71 years, who were assessed every eighth year in 1980/81, 1988/89, 1996/97, and 2004/05. The sample included all who had answered at least once, and for each assessment the sample was completed with new individuals in the age span 16–23 years, and those who were older than 71 years were removed. The surveyed individuals were invited by letter to take part in the survey. Professional interviewers from Statistics Sweden conducted face-to-face interviews, usually at the respondents’ homes. Since 1979, there have been four main themes in the SALLS: social relations, work, health and the physical environment. Certain questions about health, employment, economic resources, working environment, education and housing are repeated every year in order to provide consistent information on important background variables, e.g., self-reported health, socioeconomic conditions and family type. The data are not publicly available and the use and analysis of the data require permission from Statistics Sweden.

We excluded individuals who had missing values for the variables weight or height (1%), or physical activity. Individuals with missing values for education were classified as belonging to the highest educational level. The variables sex, age, marital status, birth cohort and urbanization had no missing values.

### Outcome variable

The question about anxiety was posed along with a list of other medical problems, such as diabetes, back pain and hypertension. Self-reported anxiety was assessed using the question” Do you suffer from nervousness, uneasiness, or anxiety?”. No limited time period for the problems was given. There were three possible answers:” no”, “yes mild” and “yes severe”. In the present study, those who reported severe or mild nervousness, uneasiness or anxiety were considered to have self-reported anxiety.

### Explanatory variables

To assess the longitudinal changes in different groups of age and birth cohort, three time-related variables were included: assessment period, age at the time of the assessment, and year of birth. Moreover, we included the following potentially explanatory variables for which previous studies have suggested an association with anxiety [11, 14–17]: sex, education level, urbanization, marital status, smoking, leisure time physical activity, and body mass index (BMI). These variables were measured in each survey and included in the models as time-varying covariates. Education, smoking, cohabiting, physical activity and weight/height were self-reported, and the other variables were obtained from the register of the total population.

Assessment period comprised four categories: 1980/81, 1988/89, 1996/97, 2004/05.

Age at the time of interview was categorized into the following groups, reflecting the 8-year intervals between the assessments: 16–23, 24–31, 32–39, 40–47, 48–55, 56–63, and 64–71 years. Age was centered at 42 years in order to have a reference group within the studied age interval.

Birth cohort (based on year of birth), comprised groups born in 1910–17, 1918–25, 1926–33, 1934–41, 1942–49, 1950–57, 1958–65, 1966–73, 1974–81, and 1982–89. Birth cohort was centered at 1950.

Sex: All analyses were made separately for males and females.

Education level: Education level (comparable over the entire study period) was categorized as: (1) high (theoretical high school and/or college, ≥12 years); (2) intermediate (practical high school, i.e., vocational school, 10–11 years); and (3) low (compulsory school or less, ≤9 years).

Urbanization: Residence in: (1) the three largest cities in Sweden; (2) medium-sized towns (population > 90,000); and (3) small towns (population 27,000–90,000) and rural areas.

Marital status was dichotomized as married/cohabiting and all others.

Smoking was dichotomized as (1) non-smokers, comprising never smokers, occasional smokers and former smokers (regardless of when they quit), and (2) daily smokers.

Leisure time physical activity was based on a question about how much physical activity the person does during leisure time, with five options from (1) “basically nothing” to (5) “regularly rather strenuously at least twice a week.” The five options were dichotomized as (1) more than once a week (options 4–5) and (2) no or some physical activity, at most once a week (options 1–3).

BMI was calculated as self-reported weight (kg)/height (m)^2^. Subjects with missing values for either weight or height (1%) were excluded, to be able to calculate BMI. BMI was categorized into (1) normal weight (20.0–24.9 kg/m2), (2) overweight (25.0–29.9 kg/m2), and (3) obesity (≥30.0 kg/m2).

### Statistical analysis

In the analysis, descriptive statistics were used to present the distributions of the variables (Table 1), as well as unadjusted prevalence of anxiety according to the explanatory variables. Differences/trends were considered as significant if p was <0.05. No adjustment of *p*-values was done. We tested trends by applying a method suggested by Cuzick, which was implemented in STATA as nptrend [19] (Table 2).

**Table 1:** Distribution (%) of the different variables according to sex and assessment period (longitudinal samples of the Swedish population from 1980/81, 1988/89, 1996/97, and 2004/05) in individuals aged 16–71 years

Variable	Males				Females			
	1980/81	1988/89	1996/97	2004/05	1980/81	1988/89	1996/97	2004/05
n	2728	2688	2570	2177	2770	2666	2634	2211
Age (years)								
16–23	16.3	16.3	12.8	12.7	16.2	16.0	12.0	13.1
24–31	17.6	15.5	16.5	13.1	15.9	15.8	16.5	13.0
32–39	19.6	16.1	16.5	16.6	17.0	15.8	16.6	16.2
40–47	13.2	18.1	16.1	15.2	12.3	16.8	16.1	16.1
48–55	10.6	12.4	17.9	14.5	12.9	11.1	16.5	15.3
56–63	12.4	9.5	11.3	17.1	13.0	12.2	11.0	15.4
64–71	10.3	12.1	8.9	10.8	12.7	12.3	11.3	10.9
Education level								
High	30.8	35.8	43.6	52.4	20.2	29.4	40.9	54.0
Intermediate	26.4	31.3	30.9	26.8	31.1	36.0	33.6	26.8
Low	42.8	32.9	25.5	20.8	48.7	34.6	25.5	19.2
Urbanization								
Large cities	30.8	31.0	31.2	34.3	30.5	30.0	31.4	33.5
Medium towns	31.5	33.8	36.5	36.0	32.6	34.7	37.4	36.1
Small towns	37.7	35.2	32.3	29.7	36.9	35.3	31.2	30.4
Marital status								
Married/cohabiting	65.5	64.5	64.8	64.6	68.2	67.7	67.6	65.6
All others	34.5	35.5	35.2	35.4	31.8	32.3	32.4	34.4
Smoking								
Non-smokers	65.9	73.1	81.8	86.5	68.9	71.3	75.8	81.3
Daily smokers	34.1	26.9	18.2	13.5	31.1	28.7	24.2	18.7
Leisure time physical activity								
None to once a week	67.9	64.7	61.3	54.7	75.0	70.6	64.8	51.9
More than once a week	32.1	35.3	38.7	45.3	25.0	29.4	35.2	48.1
Body mass index								
Normal	66.6	63.0	54.3	47.8	75.8	74.0	67.0	64.0
Overweight	29.2	31.9	38.7	42.3	19.8	20.9	26.2	26.3
Obesity	4.2	5.1	7.0	9.9	4.4	5.1	6.8	9.7

**Table 2:**
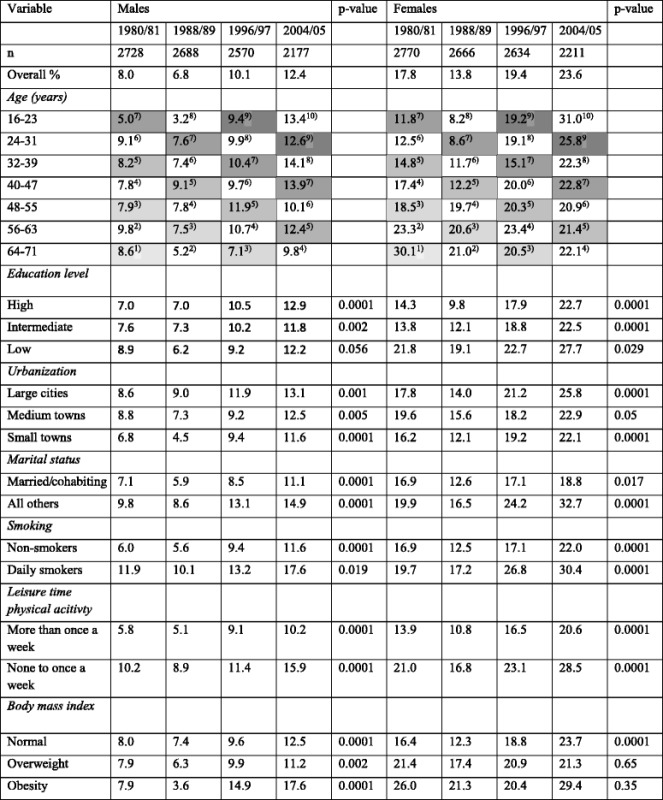
Unadjusted prevalence of self-reported anxiety (%) in individuals aged 16–71 years and tests for trends in the different explanatory variable groups, presented separately according to sex and assessment period (longitudinal samples of the Swedish population from 1980/81, 1988/89, 1996/97, and 2004/05)

Cohort:  ^2)^1918–25;  ^4)^1934–41;  ^6)^1950–57;  ^8)^1966–73;  ^10)^ 1982–89

Each birth cohort can be followed diagonally by the different grey-scales. *p*-values: Test for trend row-wise for males and females separately

A mixed logistic model with random intercepts was applied to test the change in prevalence of anxiety for age groups and cohorts in the four assessment periods (Table 3). The effect of time period does not need to be estimated for a longitudinal panel study, as age and time express the same effect. Including random slopes did not improve the model. There was a highly significant interaction between age and cohort, and this interaction was included in the models. We tested all other interactions between time and each risk factor, but none of these was significant. The unadjusted model included age, cohort, the age-by-cohort interaction, and age-squared. In a second model, adjustments were made for all explanatory variables, i.e. education level, urbanization, marital status, smoking, leisure time physical activity, and BMI. Age was centered at 42 years and cohort was centered at 1950. Odds ratios (ORs) with 95% confidence intervals (95% CIs) were calculated separately according to sex. Finally, adjusted prevalence of anxiety was calculated by using the models in Tables 3 and 4 (Table 5). Birth cohort trends (change per birth year) and age trends (change per year) were also calculated. The trends for each age group and cohort were estimated by applying a linear regression model with time as the independent variable and with the estimated proportions in Tables 3 and 4 as the dependent variable.

**Table 3:** Odds ratios (ORs) with 95% confidence intervals (95% CIs) for self-reported anxiety in males aged 16–71 years in Sweden in 1980/81–2004/05, estimated using mixed models with random intercepts

		Unadjusted model		Adjusted model	
Variable	Category	OR	95% CI	OR	95% CI
Fixed effects					
Rate of change					
OR by age^1^	Age centered at 42 years	1.04	1.03–1.05	1.05	1.04–1.06
Agec*cohortc		0.9994	0.998–1.000	0.9993	0.9987–1.00002
Agec-squared		0.998	0.997–0.999	0.998	0.997–0.999
OR by cohort^2^	Cohort centered at 1950	1.03	1.02–1.04	1.04	1.03–1.05
Education level	High			1	Reference
	Intermediate			1.01	0.79–1.28
	Low			0.96	0.74–1.23
Urbanization	Large cities			1	Reference
	Medium towns			0.91	0.72–1.16
	Small towns			0.68	0.53–0.88
Marital status	Married/cohabiting			1	Reference
	All others			2.03	1.65–2.51
Smoking	Non-smokers			1	Reference
	Daily smokers			1.71	1.38–2.14
Leisure time physical activity	More than once a week			1	Reference
	None to once a week			1.67	1.39–2.02
Body mass index	Normal			1	Reference
	Overweight			0.88	0.72–1.09
	Obesity			1.23	0.85–1.79
Variance components		Variance	Standard error	Variance	Standard error
	Var (cons)	3.33	0.38	3.02	0.35

Agec, age-centered; cohortc, cohort-centered

Agec*cohortc represents interaction

OR, odds ratio. CI, confidence interval

^1^OR by increasing age and ^2^OR by later/younger cohort

Unadjusted model included agec, cohortc, the age-by-cohort interaction, and age-squared

Adjusted model also included education level, urbanization, marital status, smoking, leisure time physical activity, and body mass index

**Table 4:** Odds ratios (ORs) with 95% confidence intervals (95% CIs) for self-reported anxiety in females aged 16–71 years in Sweden in 1980/81–2004/05, estimated using mixed models with random intercepts

		Unadjusted model		Adjusted model	
Variable	Category	OR	95% CI	OR	95% CI
Fixed effects					
Rate of change					
OR by age^1^	Age centered at 42 years	1.04	1.03–1.05	1.05	1.04–1.06
Agec*cohortc		0.998	0.997–0.999	0.998	0.997–0.999
Agec-squared		0.998	0.997–0.999	0.998	0.997–0.999
OR by cohort^2^	Cohort centered at 1950	1.03	1.02–1.04	1.03	1.02–1.04
Education level	High			1	Reference
	Intermediate			0.93	0.76–1.15
	Low			1.29	1.04–1.59
Urbanization	Large cities			1	Reference
	Medium towns			0.95	0.78–1.15
	Small towns			0.80	0.65–0.98
Marital status	Married/cohabiting			1	Reference
	All others			1.81	1.53–2.14
Smoking	Non-smokers			1	Reference
	Daily smokers			1.61	1.35–1.93
Leisure time physical activity	More than once a week			1	Reference
	None to once a week			1.43	1.24–1.65
Body mass index	Normal			1	Reference
	Overweight			1.04	0.87–1.25
	Obesity			1.31	0.97–1.77
Variance components		Variance	Standard error	Variance	Standard error
	Var (cons)	3.39	0.29	2.98	0.27

Agec, age-centered; cohortc, cohort-centered

Agec*cohortc represents interaction

*OR* odds ratio, *CI* confidence interval

^1^OR by increasing age and ^2^OR by later/younger cohort

Unadjusted model included agec, cohortc, the age-by-cohort interaction, and age-squared

Adjusted model also included education level, urbanization, marital status, smoking, leisure time physical activity, and body mass index

**Table 5:**
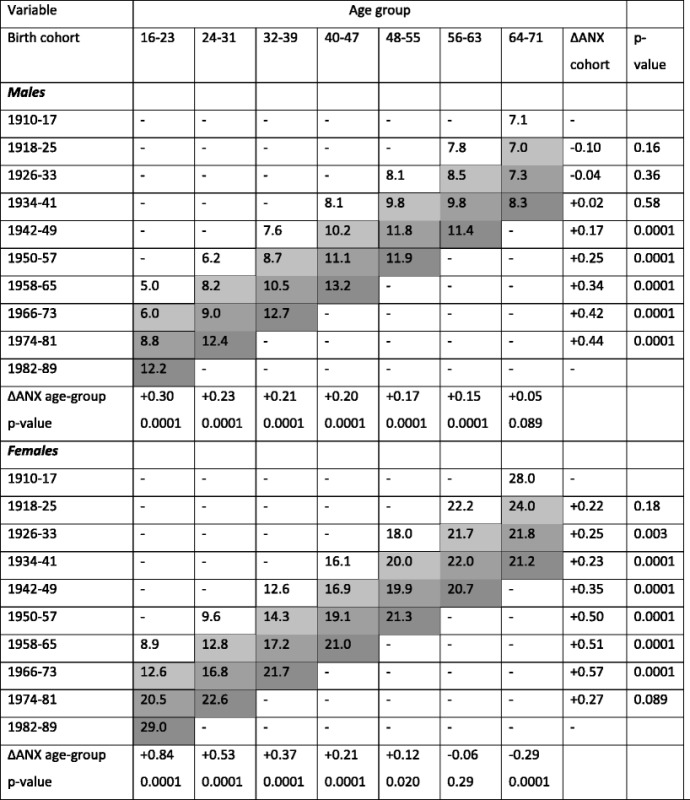
Adjusted prevalence (%) of self-reported anxiety based on the adjusted models in Tables 3 and 4, and annual change in anxiety (ΔANX per year by age and cohort, test of trend) in males and females aged 16–71 years, presented according to age, cohort (birth year), and assessment period (longitudinal samples of the Swedish population from 1980/81, 1988/89, 1996/97, and 2004/05)

Colour code for assessment periods: 1980/81, ,  and .

*ΔANX* annual change in anxiety prevalence

We analysed non-response carefully, and found differences in distributions between responders and non-responders. We have taken this fact into consideration by including the post stratification variables (age, marital status, urbanization and education) in the models. By including the post-stratification variables, no weights are necessary, according to Nordberg [20]. Nordberg means that this way of compensation for missing data might even be better than calculating weights based on the same variables. In an additional analysis, we used sampling weights.

STATA version 13 [21] was used for the statistical analyses.

### Ethics

This study was approved by the ethics committee in Stockholm (approval no. 12/2000).

## Results

The distribution of the different explanatory variables is presented, separately according to sex and assessment period, in Table 1. During the 25-year study period, education level and urbanization tended to increase, as well as non-smoking, leisure time physical activity, and overweight/obesity. Marital status did not change markedly.

In Table 2, unadjusted prevalence of anxiety by the different explanatory variables is presented separately according to sex and assessment period. Anxiety was more common in females, and the overall prevalence of anxiety increased in both males and females during the study period. In 2004/05, almost one fourth of the females reported anxiety. In order to show the trends in anxiety over time in each birth cohort, the ten different birth cohorts are marked with numbers 1–10 in superscript in Table 2. Reading the numbers 1–10 diagonally shows that later/younger birth cohorts reported increased prevalence of anxiety during the study period, while the earliest/oldest birth cohorts did not. Reading the table horizontally reflects a time trend of increased prevalence of anxiety in all age groups except from males 64–71 years and females 56–71 years.

Unadjusted tests for longitudinal trends (*p*-values) in anxiety in covariate subgroups are also shown in Table 2. The prevalence of anxiety increased in almost all subgroups, except from overweight and obese females. However, baseline prevalence in 1980/81 was higher in overweight/obese females than in normal weight.

The results for the mixed models are presented in Table 3 (males) and 4 (females). In both sexes, there was a significantly increased OR for anxiety by age (centered at 42 years) as well as by cohort (centered at the 1950 birth cohort), reflecting increased anxiety by older age and by later/younger cohort. In males, the adjusted OR for anxiety by age was 1.05 (95% CI: 1.04–1.06) per year. The adjusted OR by birth cohort was 1.04 (95% CI: 1.03–1.05), per cohort year. The figures were similar in females.

There was a statistically significant interaction between age and cohort in females (OR 0.998, 95% CI 0.997–0.999), and a borderline significant interaction in males, indicating that the effect of age differed between cohorts, i.e. stronger increases in anxiety in younger birth cohorts. Age-squared was also significant, reflecting the non-linear association.

Tables 3 and 4 also show the relationships between anxiety and covariates. In both males and females, increased anxiety was associated with non-married status, smoking and low physical activity. In females, increased anxiety was also associated with lower education level and living in a large city.

Table 5 presents adjusted prevalence of anxiety according to cohort and age group, based on the models in Tables 3 and 4. This table also shows annual changes in anxiety in the studied birth cohorts and age groups. By reading the table vertically, one can see the time trends of anxiety between 1980/81 and 2004/05 in the different age groups. In males, anxiety increased significantly over time in all age groups except from the oldest aged 64–71 years. In females, anxiety increased significantly in all age groups except from those aged 56–71 years. In the youngest age group 16–23 years, there was a dramatic increase in anxiety prevalence in both males (from 5.0 to 12.2%) and females (from 8.9 to 29.0%). By reading the table horizontally, one can see significantly increased anxiety prevalence in all birth cohorts except from birth cohorts 1941 and earlier/older in males, and birth cohorts 1925 and earlier/older in females.

### Non-response

The non-response rate in the initial sample was about 20% and it increased over the years to about 25%. We have compared estimates from the entire sample with the longitudinal part. These estimates were close to each other. Therefore, we think the sample is representative. Furthermore we have compared the response pattern in the variables (sex, age, marital status, urbanization) from the register of the total population, and the response pattern was similar over the years, e.g. women had always higher response than men over the years. As there were differences in distributions between responders and non-responders, we included the post stratification variables (age, marital status, urbanization and education) in the models. Using sampling weights gave about the same results.

## Discussion

The main finding of this longitudinal study between 1980/81 and 2004/05, was the increasing prevalence of self-reported anxiety over time, in all age groups except from the oldest age groups. There was a dramatic increase of anxiety during the 25-year long follow-up time especially in the youngest group 16–23 years, with more than a three-fold increase in females, and a 2.5-fold increase in males, after adjustments for covariates. In contrast, in females, the prevalence of anxiety declined significantly in the oldest age group 64–71 years. Moreover, there was a trend of increased prevalence of anxiety by increasing age between 16 and 23 years and 64–71 years, in almost all birth cohorts except from the oldest/earliest cohorts, where there was no clear trend.

These results may have a large impact on public health and healthcare demands. According to a Swedish study, 20% of those who visited a family physician in 2011 received help for mental problems, but only 7% received a psychiatric diagnose [4]. This supports that there is a great suffering in mental illness even without psychiatric diagnoses. The results could probably be explained by fear of stigmatisation or that individuals with mental problems are poorly identified. Anxiety disorders often start in adolescence and are therefore important and possible to prevent [3, 22, 23]. Increased self-reported anxiety in young individuals are in line with a recent international review of the literature, which shows that recent cohorts of adolescents, especially females, experience increased mental illness compared to previous birth cohorts [7, 11]. Early interventions are therefore crucial and schools, including high schools and colleges, play an important role, in addition to familial and socioeconomic factors. Increased availability of psychosocial interventions in health care centers, such as psychologists, could also be valuable. Our recently published study of the same study population showed a trend of deteriorating self-rated health in young adults, between 1980 and 2005, which further points out the vulnerability of this young age group [24].

Although our results do not allow us to draw any causal inferences, there are several potential mechanisms behind our findings. Today, the entrance to the labour market has been delayed to a higher age, which is due to increased educational demands at the labour market and unemployment in young adults [8]. Such mechanisms may lie behind our findings of increased reporting of anxiety in young individuals [8, 25, 26]. In the 1990s, Sweden underwent a financial crisis which, among other things, resulted in increased unemployment rates and poverty in youths while older generations experienced a more favourable development [5, 8]. During the beginning of the twenty-first century, many European countries have experienced a similar development, and other studies have also reported increased prevalence rates of psychiatric illness during economic downturns [27]. In age group 16–23 years, there was a higher increase in anxiety prevalence between assessment 2–3 and 3–4 than between the first two assessments, which could mirror these economic changes in the society (Table 5).

The increased prevalence of anxiety over time was seen in both young females and males and the absolute prevalence was higher in the young women, which is in line with previous research showing that anxiety disorders are more common in females than in males [7, 11, 28]. It has previously been discussed whether girls and young women are more stressed and anxious of their school performance, as well as more concerned about their physical appearance [10, 29]. The increased prevalence of anxiety by increasing age in most birth cohorts could partly be explained by the natural anxiety psychopathology as people age, as anxiety disorders are highly chronic and individuals usually don’t recover [30]. However, the median age of onset for anxiety disorders is around 11 years, and the 75th percentile of the age-of-onset is 21 years [3]. Consequently, the major part of the individuals has already become affected by their anxiety in the first age group 16–23 years old.

It is important to note that the potential stigma associated with mental illness has changed quite dramatically over time and, today, many people are more aware of and likely to discuss their psychiatric symptoms openly, even if stigmatisation still exists. People also have a better insight and are more informed about mental illness. The change in attitudes and higher awareness of mental health over recent years may have influenced changes of patterns of reporting of anxiety and the readiness to report symptoms [7]. It is possible that this change in stigma has been more prominent in the youngest cohorts. However, the oldest age group showed a declining prevalence of anxiety in females, which supports our interpretation that the changes are not merely caused by differences in stigma over time. Moreover, irrespective of whether the reported results are “true” changes of the prevalence of anxiety or only changes in patterns of reporting, the increased self-reported anxiety is likely to increase the demand for health care.

### Limitations and strengths

This study has some important limitations. The study is based on self-reported survey data about perceived nervousness and anxiety, and we had no objective measurements of anxiety. The individuals’ answers reflect how they interpret their feelings when they answer the survey. Consequently, there may be overestimation as well as underestimation of the reporting of anxiety, and it is possible that some answers were misclassified. However, the aim of the study was to study the longitudinal changes of self-reported anxiety, and the survey question was the same in all four assessments, so this will probably not affect the conclusions. The survey question has also been reported to be a strong predictor of premature mortality and psychiatric disorders [13], which supports that it is a valid indicator of psychiatric as well as somatic health. Another possible limitation is that the non-response rate was 20–25%. However, in analyses of non-responders, we found that the pattern of non-response was similar according to sex, age, marital status, urbanization, and income over time. As non-response has been reported to be associated with poorer mental health [31], it is likely that the prevalence of anxiety is higher among non-responders, although this bias would be similar in all assessments. Finally, even if we were able to control for several potential confounding factors, it is possible that there are residual confounding factors, for example immigration status, unemployment, alcohol and drugs [32].

The limitations in this study are balanced by several strengths, which include a long follow-up period (25 years) and that the SALLS is one of the most comprehensive national surveys [18]. The study population is representative of the entire Swedish population, as it is a random sample with a longitudinal “panel” with repeated measurements, drawn from the Total Population Register. The longitudinal mixed model made it possible to distinguish changes over time within individuals (age effects) from differences among individuals at baseline (cohort effects). The interviews were mainly conducted in the respondents’ homes by well-trained interviewers and the reliability of the survey questions has been estimated by re-interviewing a subsample of the participants (test-retest method) [33].

## Conclusions

In summary, we found increased prevalence of self-reported anxiety over time between 1980/81 and 2004/05 in all age groups except from the oldest, which indicates increased suffering for a large part of the population. Clinical efforts should focus particularly on young females (16–23 years), where almost one third experienced anxiety in 2005, and whose reporting of anxiety has increased dramatically. It is important that health care centres provide psychosocial interventions, e.g. psychologists, for patients with mental illness. Perhaps suggesting support groups/ interventions at the high school or college level could also be a step in the right direction. Future research should preferably focus on interventions aiming to improve mental health in young adults. Moreover, studies designed to disentangle the reasons of increased perceived anxiety are needed.

## References

[1] World Health Organization.The World Health report (2001). Mental health, new understanding, new hope.

[2] Murray CJ, Lopez AD (1997). Global mortality, disability, and the contribution of risk factors: Global Burden of Disease Study. Lancet.

[3] Kessler RC, Berglund P, Demler O, Jin R, Merikangas KR, Walters EE (2005). Lifetime prevalence and age-of-onset distributions of DSM-IV disorders in the National Comorbidity Survey Replication. Arch Gen Psychiatry.

[4] Asbring N, Dal H, Ohrling M, Dalman C (2014). One in five who visited a health center received help for mental illness. But only 7 percent were given a psychiatric diagnosis, as shown in a database study in Stockholm. Lakartidningen.

[5] Bremberg S (2006). Ungdomar, stress och psykisk ohälsa (Adolescents, stress and mental illness). Swedish.

[6] Pirraglia PA, Hampton JM, Rosen AB, Witt WP (2011). Psychological distress and trends in healthcare expenditures and outpatient healthcare. Am J Manag Care.

[7] Bor W, Dean AJ, Najman J, Hayatbakhsh R (2014). Are child and adolescent mental health problems increasing in the 21st century? A systematic review. Aust N Z J Psychiatr.

[8] Kosidou K, Magnusson C, Mittendorfer-Rutz E, Hallqvist J, Hellner Gumpert C, Idrizbegovic S (2010). Recent time trends in levels of self-reported anxiety, mental health service use and suicidal behaviour in Stockholm. Acta Psychiatr Scand.

[9] Sigfusdottir ID, Asgeirsdottir BB, Sigurdsson JF, Gudjonsson GH (2008). Trends in depressive symptoms, anxiety symptoms and visits to healthcare specialists: a national study among Icelandic adolescents. Scand J Public Health.

[10] Wiklund M, Malmgren-Olsson EB, Ohman A, Bergstrom E, Fjellman-Wiklund A (2012). Subjective health complaints in older adolescents are related to perceived stress, anxiety and gender - a cross-sectional school study in Northern Sweden. BMC Public Health.

[11] Keyes KM, Nicholson R, Kinley J, Raposo S, Stein MB, Goldner EM (2014). Age, period, and cohort effects in psychological distress in the United States and Canada. Am J Epidemiol.

[12] Smith RP, Larkin GL, Southwick SM (2008). Trends in U.S. emergency department visits for anxiety-related mental health conditions, 1992-2001. J Clin Psychiatr.

[13] Ringback Weitoft G, Rosen M (2005). Is perceived nervousness and anxiety a predictor of premature mortality and severe morbidity? A longitudinal follow up of the Swedish survey of living conditions. J Epidemiol Community Health.

[14] Lasser K, Boyd JW, Woolhandler S, Himmelstein DU, McCormick D, Bor DH (2000). Smoking and mental illness: A population-based prevalence study. JAMA.

[15] Rajmil L, Herdman M, Ravens-Sieberer U, Erhart M, Alonso J, European Kg (2014). Socioeconomic inequalities in mental health and health-related quality of life (HRQOL) in children and adolescents from 11 European countries. Int J Public Health.

[16] Luppino FS, de Wit LM, Bouvy PF, Stijnen T, Cuijpers P, Penninx BW (2010). Overweight, obesity, and depression: a systematic review and meta-analysis of longitudinal studies. Arch Gen Psychiatry.

[17] Goodwin RD (2003). Association between physical activity and mental disorders among adults in the United States. Prev Med.

[18] StatisticsSweden. Living Conditions. Appendix 16. The Swedish survey of living conditions. Design and methods. http://www.scb.se/contentassets/0d073cdda6a345ac8edc975fa20ad5cb/appendix16.pdf. Stockholm: 1996.

[19] Cuzick J (1985). A Wilcoxon-type test for trend. Stat Med.

[20] Nordberg L. Generalized Linear Modeling of Sample survey data. J Off Stat. 1989:223–39.

[21] StataCorp (2011). Stata Statistical Software: Release 12.

[22] Egger HL, Angold A (2006). Common emotional and behavioral disorders in preschool children: presentation, nosology, and epidemiology. J Child Psychol Psychiatr.

[23] Mesman J, Koot HM (2001). Early preschool predictors of preadolescent internalizing and externalizing DSM-IV diagnoses. J Am Acad Child Adolesc Psychiatry.

[24] Johansson SE, Midlov P, Sundquist J, Sundquist K, Calling S (2015). Longitudinal trends in good self-rated health: effects of age and birth cohort in a 25-year follow-up study in Sweden. Int J Public Health.

[25] Lo CC, Cheng TC (2014). Race, unemployment rate, and chronic mental illness: a 15-year trend analysis. Soc Psychiatry Psychiatr Epidemiol.

[26] Sundquist K, Ahlen H (2006). Neighbourhood income and mental health: a multilevel follow-up study of psychiatric hospital admissions among 4.5 million women and men. Health Place.

[27] Christodoulou NG, Christodoulou GN (2013). Financial crises: impact on mental health and suggested responses. Psychother Psychosom.

[28] Zahn-Waxler C, Shirtcliff EA, Marceau K (2008). Disorders of childhood and adolescence: gender and psychopathology. Annu Rev Clin Psychol.

[29] West P, Sweeting H (2003). Fifteen, female and stressed: changing patterns of psychological distress over time. J Child Psychol Psychiatr.

[30] Bruce SE, Yonkers KA, Otto MW, Eisen JL, Weisberg RB, Pagano M (2005). Influence of psychiatric comorbidity on recovery and recurrence in generalized anxiety disorder, social phobia, and panic disorder: a 12-year prospective study. Am J Psychiatry.

[31] Mars B, Cornish R, Heron J, Boyd A, Crane C, Hawton K, et al. Using Data Linkage to Investigate Inconsistent Reporting of Self-Harm and Questionnaire Non-Response. Arch Suicide Res. 2016:1–29. 10.1080/13811118.2015.1033121 PMC4841016 26789257

[32] Marmorstein NR (2009). Longitudinal associations between alcohol problems and depressive symptoms: early adolescence through early adulthood. Alcohol Clin Exp Res.

[33] Wärneryd B. Återintervjustudie i undersökningen av levnadsförhållanden 1989 (ULF) (Living conditions. Reinterview in ULF 1989) Appendix 12. Swedish. Stockholm: Statistics Sweden; 1991.

